# Metabolic profiling and *in vitro*-*in vivo* extrapolation of furathiocarb in mammalian hepatic microsomes

**DOI:** 10.1016/j.toxrep.2022.03.030

**Published:** 2022-03-29

**Authors:** Khaled Abass, Petri Reponen, Walaa F. Alsanie, Arja Rautio, Olavi Pelkonen

**Affiliations:** a Arctic Health, Faculty of Medicine, University of Oulu, P.O. Box 7300, FI-90014, Finland; b Pharmacology and Toxicology Unit, Research Unit of Biomedicine, University of Oulu, P.O. Box 5000, FI-90014 Oulu, Finland; c Department of Pesticides, Menoufia University, P.O. Box 32511, Egypt; d Department of Clinical Laboratory Sciences, The Faculty of Applied Medical Sciences & Centre of Biomedical Sciences Research (CBSR), Taif University, Saudi Arabia; e Thule Institute, University of the Arctic, FI-90014 Oulu, Finland

**Keywords:** Pesticides, Toxicokinetics, CYP450, *In vitro* metabolism, Species differences, Risk assessment

## Abstract

Furathiocarb is a carbamate insecticide found in marine ecosystems as well as river water and sediments. The aim of this study was to characterize species differences in the *in vitro* metabolism of furathiocarb in seven mammalian species (human, monkey, minipig, rat, mouse, dog, rabbit) analyzed by LC-TOF-MS/MS, in order to provide qualitative and quantitative chemical-specific data to enhance toxicological risk assessment. Furathiocarb was mainly biotransformed to carbofuran metabolic pathway *via* (N-S) bond-cleavage. Two hydroxylated and sulfoxidated metabolites of furathiocarb were also detected (oxidation pathway). No unique human metabolites were detected. The carbofuran metabolic pathway was more predominant than the furathiocarb oxidation pathway in all species studied; differences based on hepatic clearance rates (*CL*_*H*_), were up to 9.4-fold in monkey and 7-fold in rats, while it was 4.3-fold in human. Animal to human differences in the carbofuran pathway are within the default toxicokinetic uncertainty factor, except for mouse (3.9-fold). Our findings on metabolic profiling and *in vitro*-*in vivo* extrapolations are helpful for the interpretation of toxicological findings and chemical risk assessment of furathiocarb.

## Highlights

• We studied comparative *in vitro* hepatic biotransformation of furathiocarb.
• Human, monkey, minipig, rat, mouse, dog and rabbit hepatic microsomes were studied.
• No unique human or experimental animal metabolites were detected.
• The carbofuran pathway was more rapid than the oxidation pathway in all species.
• Quantitative interspecies differences observed in metabolite formations and kinetics.

## Graphical Abstract

**Figure:**
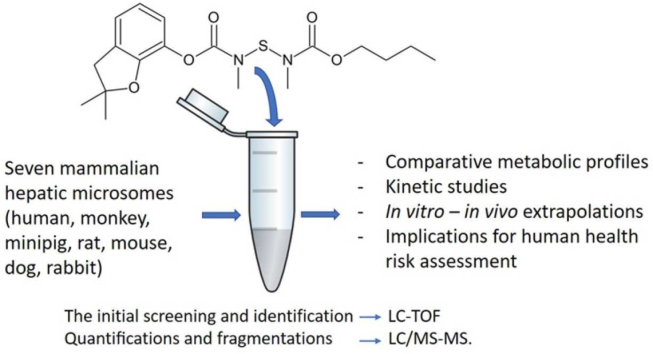

## 1 Introduction

Furathiocarb [(2,2-dimethyl-3 H-1-benzofuran-7-yl) N-[butoxycarbonyl(methyl)amino]sulfanyl-N-methylcarbamate] belongs to the carbamate class of pesticides. Furathiocarb is a pro-insecticide that forms predominantly biologically active metabolites, carbofuran and 3-hydroxy carbofuran [25]. Carbofuran is a more potent AChE inhibitor than furathiocarb due to furathiocarb-bulky carbamoyl moiety, which decreases rat brain AChE inhibition constants by 120-fold [36]. Furathiocarb degrades very slowly (10 000 days or more), and therefore, persists in the environment and may have adverse effects on the ecological environment and human health [30]. Furathiocarb and its metabolite, carbofuran, are included in different monitoring programs for pesticide residues in food and in the environment [18], [19], [22], [46]. These compounds were not found in quantifiable concentrations in plant products during the official control activities carried out by EU Member States, Iceland, and Norway in 2016 [13]. Even though furathiocarb is currently not approved for use in the EU, it was detected in both fat and muscle tissue of loggerhead turtles, widely used as bioindicators of contamination in marine ecosystems, on the Eastern coast of Spain [31]. Carbofuran was found at higher concentrations in water and sediments of the Turia and Jucar rivers in Eastern Spain [10].

Investigation of interspecies differences of pesticides metabolism is required by EU regulation 1107/2009 [14], [15], [16] to guarantee similar metabolic profiles between humans and the animal species used in safety studies. Studying qualitative/quantitative similarities and differences in pesticides biotransformation between human and other mammalian species allows the selection of the most appropriate animal model for *in vivo* toxicology studies [44].

Little is known about furathiocarb biotransformation in humans and mammals. Furathiocarb and its metabolites, carbofuran, 3–hydroxycarbofuran and 3–ketocarbofuran, were reported in animals [38]. Carbofuran and 3–hydroxycarbofuran were detected in plasma and urine of male Sprague–Dawley rats following dermal treatment with furathiocarb [27]. Furathiocarb was metabolized only to the more toxic carbofuran in an *in vitro* dermal penetration study with rat abdominal skin using the static diffusion cell [26]. Carbofuran and 3-hydroxycarbofuran were detected in liver, while only carbofuran was detected in kidney after dermal treatment of Sprague-Dawley rats with furathiocarb [28].

Cytochrome P450 enzymes (CYP450) are the key enzymes responsible for the metabolism and biotransformation of a wide range of endogenous substrates and xenobiotics, including pesticides, in mammals including humans [1], [21], [35]. Metabolic pathways of furathiocarb in mammals have not been investigated in detail. Furthermore, no information is available on the mammalian, including human, hepatic enzyme kinetics of furathiocarb. Quantitative toxicokinetic data in human and animal species are pivotal for further development of chemical-speciﬁc adjustment factors (CSAFs) [12], [45]. Therefore, the aim of the current study was to quantitatively investigate furathiocarb metabolic pathways in *in vitro* hepatic microsomes of seven mammalian species, including human, *in vitro-*to*-in vivo* extrapolation as well as its implications for human health risk assessment.

## 2 Materials and methods

Additional detailed information on items of Materials and Methods are available as supplementary materials.

### 2.1 Chemicals

Furathiocarb and its metabolites, except the hydroxylated and sulfoxidated-furathiocarb, were purchased from ChemService (West Chester, PA). Solvents, HPLC-grade, were purchased from Rathburn (Walkerburn, UK) and Labscan (Dublin, Ireland). Other chemicals were purchased from Sigma Chemical Company (St. Louis, MO, USA) and were of the highest purity available.

### 2.2 Human liver homogenates and mammalian liver microsomes

The Ethics Committee of the Medical Faculty of the University of Oulu, Finland approved the collection of human hepatic surplus from organ donors. Detailed characteristics of the collected samples are presented in supplementary materials. Male Sprague-Dawley rat, DBA/2 mouse, New Zealand white rabbit, Göttingen minipig, Cynomolgus monkey, and Beagle dog liver samples were obtained after approval of the Ethics Board of the Experimental Animal Center of the University of Oulu, Finland. All enzyme activity characterizations as well as comparative incubations of furathiocarb metabolism with different mammalian hepatic microsomes were performed within 12 months (Detailed information are available as supplementary materials).

### 2.3 *In vitro* assay of furathiocarb metabolites and kinetic parameters

The standard incubation mixture, 200 µl of 0.1 M phosphate buffer (pH 7.4), contained 100 µM furathiocarb, 1 mM NADPH, and 0.15 mg pooled microsomal protein. The reaction was terminated by with 600 µl of ice-cold acetonitrile after 20, 40, and 60 min incubation at + 37 °C.

Human liver homogenate incubations were employed to measure the production of potential metabolites. The incubation mixture (ﬁnal volume of 200 µl of 0.1 M phosphate buffer) contained 100 µM furathiocarb, 40 µl of human liver homogenate, 1 mM glutathione, 5 mM UDPGA, 1.2 mM PAPS, and 1 mM NADPH. Incubations and analytical methods were similar to the microsomal preparations.

Enzyme kinetic parameters were measured in microsomal preparations (furathiocarb final concentrations 2.5 – 300 µM). The conversion percentage at the concentration of 2.5 µM in human hepatic microsomes was 5.5%. Samples were analyzed by LC-MS-MS and kinetic parameters were calculated using GraphPad Prism 9 Software (San Diego, CA) by nonlinear regression (Michaelis-Menten, kinetics). Detailed methodologies are reported as supplementary materials.

### 2.4 Mass spectrometry

**Fig. 1:**
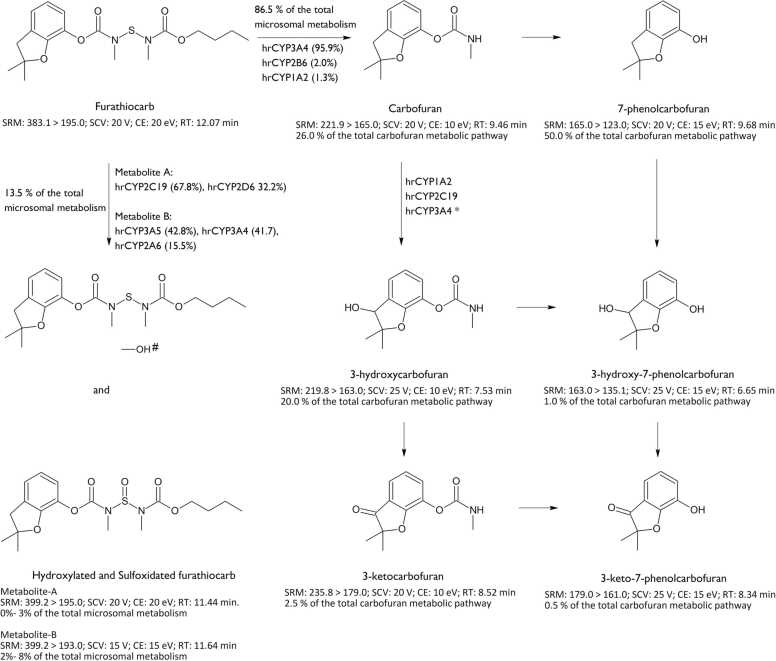
Analytical parameters and metabolic pathways of furathiocarb in human hepatic microsomes and recombinant CYP-enzymes. 3-hydroxycarbofuran and 3-hydroxy-7-phenolcarbofuran were quantified as the protonated dehydrated molecule [M−H_2_O+H]^+^ due to significant in-source fragmentation. Carbaryl was used as an internal standard (SRM: 202.00 ˃ 145.00; SCV: 25 V; CE: 15 eV; RT: 9.8 min). SRM; selected reaction monitoring; SCV: sample cone voltage (V); CE: collision energy (eV); RT: retention time. *Hydroxylation may take place on the carbamate N-methyl group, on an alkyl substituent, or on the aromatic ring itself. The percentages of metabolite formations are based on furathiocarb biotransformation (150 µM) in pooled human hepatic microsomes from 10 donors. The contribution of human recombinant CYP are based on our findings (Abass et al. submitted). *The role of recombinant enzymes in 3-OH-carbofuran formation is based on [43].

Present and accurate mass measurements were carried out by Micromass LCT - TOF-MS (Micromass, Altrincham, UK) equipped with a Z-Spray ionization source. Micromass Quattro II triple quadrupole instrument equipped with a Z-spray ionization source was employed for the quantification and fragmentation measurements. Multiple reaction monitoring (MRM), collision energies, and sample cone voltages for metabolites and the internal standard are described in Fig. 1.

### 2.5 *In vitro - in vivo* extrapolations

*In vivo* hepatic clearances were extrapolated based on *in vitro* data, microsomal protein amount per gram of liver measurements as well as other published values [34], [6], [8]. Pharmacokinetic parameters were estimated based on:

1. *In vitro* intrinsic clearance
  $$\mathrm{Clint},\mathrm{LM}\left[\frac{\frac{\mathrm{ml}}{\min}}{\mathrm{mg}}\mathrm{protein}\right]=V\max/Km\;$$
2. Hepatic intrinsic clearance
  $$\mathrm{CL}\mathrm{int},\mathrm{whole liver}\;\left[\frac{\mathrm{ml}}{\min}\right]=\;\mathrm{CL}\mathrm{int},\mathrm{LM}\;\left[\frac{\frac{\mathrm{ml}}{\min}}{\mathrm{mg}}\mathrm{protein}\right]*\mathrm{MPPGL}*\mathrm{liver weight}$$
3. Hepatic clearance

$$\mathrm{CL}H\;\left[\frac{L}{\min}\right]=\mathrm{CL}\mathrm{int},\;\mathrm{whole}\;\mathrm{liver}\;\left[\frac{L}{\min}\right]*QH\;/\;(\mathrm{CL}\mathrm{int},\;\mathrm{whole}\;\mathrm{liver}\;\left[\frac{L}{\min}\right]\;+\;QH)\;$$

## 3 Results and discussion

### 3.1 Profiles of furathiocarb metabolites in hepatic microsomes

Metabolic profiles of furathiocarb in hepatic microsomes from various mammalian species were examined by incubation of pooled hepatic microsomes (0.15 mg protein/ml) of human (humanLM), rat (ratLM), mouse (mouseLM), dog (dogLM), rabbit (rabbitLM), minipig (minipigLM), and monkey (monkeyLM) with various concentrations of furathiocarb. The initial screening and identification of the metabolites by accurate mass measurements were carried out using a LC-TOF and the quantiﬁcation and fragmentation measurements were carried out by LC/MS-MS and described in detail in our report (Abass et al. submitted). Briefly, upon incubation in seven mammalians hepatic microsomes, including pooled human hepatic microsomes, furathiocarb was metabolized to eight phase I metabolites. Six metabolites, representing the carbofuran pathway (carbofuran, 3-hydroxycarbofuran, 3-ketocarbofuran, 3-keto-7-phenolcarbofuran, 3-hydroxy-7-phenolcarbofuran, and 7-phenolcarbofuran) were identified with the help of authentic standards. Two unidentiﬁed metabolites, representing the oxidation pathway, are probably either hydroxylated or sulfoxidated derivatives of furathiocarb. Analytical parameters (The MRM, collision energies, and sample cone voltages) and metabolic pathways of furathiocarb in pooled human hepatic microsomes are presented in Fig. 1.

Limited data is available on the metabolism of furathiocarb in mammals. Furathiocarb was found in human gastric contents by TLC, GC/MS, and GC NPD [24]. Only carbofuran was detected in a furathiocarb dermal penetration study measured by rat abdominal skin, suggesting furathiocarb is biotransformed into carbofuran while passing through the skin [26]. However, detailed kinetics studies using human cDNA-expressed cytochrome P450s, correlation analysis of marker activities in individual human hepatic microsomes, as well as chemical inhibition studies showed that the two proposed hydroxylated/sulfoxidated metabolites are not the same since they were generated by different CYP isoforms. CYP2C19 and CYP2D6 mediated the formation of the metabolite detected at RT 11.44 min (hydroxylated or sulfoxidated metabolite, named metabolite A). While CYP3A5, CYP3A4, and CYP2A6 mediated the formation of the hydroxylated or sulfoxidated metabolite detected at RT 11.64 min (named metabolite B) (Abass et al. submitted). Our findings showed that the main metabolic pathways of furathiocarb in mammalian hepatic microsomes *in vitro* were the hydroxylation and sulfoxidation of furathiocarb (metabolite A and B) and the cleavage of the nitrogen–sulfur bond to give the carbofuran metabolic pathway.

### 3.2 Disappearance of furathiocarb in hepatic microsomal incubations

**Fig. 2:**
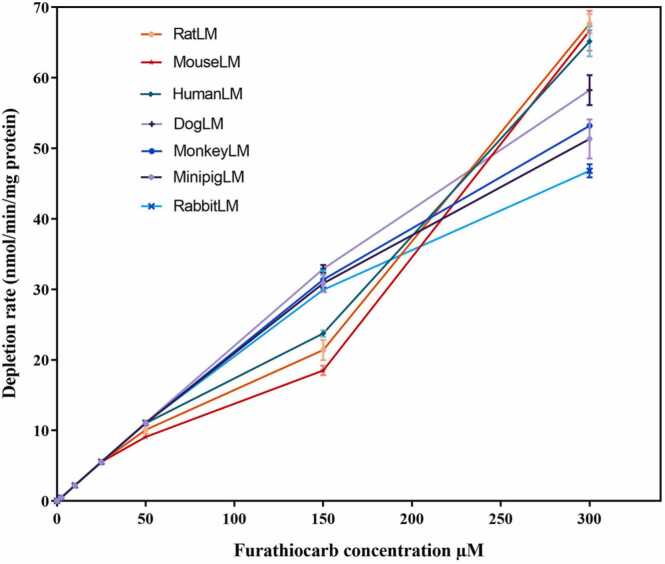
The concentration-dependent depletion rate of furathiocarb in hepatic microsomes of various species. The standard incubation mixture of 200 µl of 0.1 M phosphate buffer (pH 7.4) contained furathiocarb ﬁnal concentrations 2.5–300 µM, 0.15 mg pooled liver microsomal protein, and 1 mM NADPH. All incubations were carried out in triplicate.

The concentration-dependent depletion curves as quantiﬁed by triple quadrupole mass spectrometry showed that furathiocarb depletion in all tested hepatic microsomes was enzyme-mediated. The depletion rates of furathiocarb in hepatic microsomes from various species were different. RatLM, mouseLM, and humanLM displayed the highest final depletion rates, but this was based on a change in depletion rate between 150 and 300 µM. It is possible that a new CYP enzyme with a higher *K*_*m*_ value catalyzed this faster depletion at higher concentrations. The depletion in rabbitLM was the slowest as depicted in Fig. 2. However, the differences were not particularly large.

### 3.3 Formation of metabolites as a function of incubation time

**Fig. 3:**
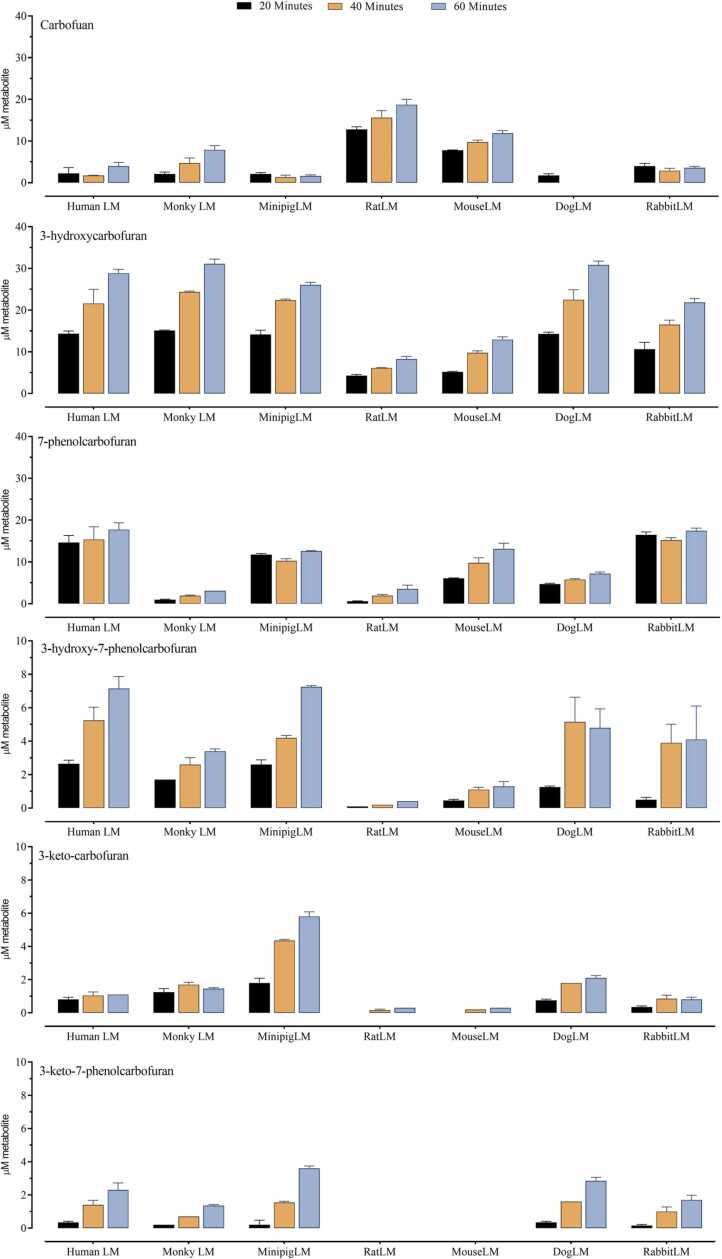

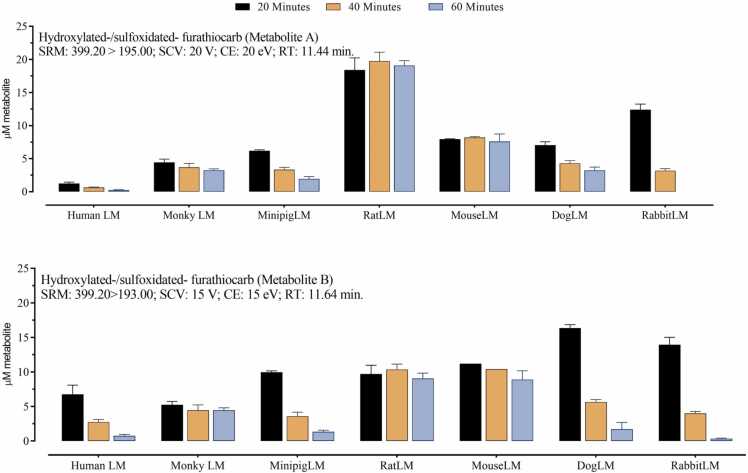
Furathiocarb metabolites formation as a function of incubation time in the presence of hepatic microsomes of 7 mammalian species. Columns represent the means of three separate measurements and the error bars represent S.D. A) carbofuran, 3-hydroxycarbofuran, 3-ketocarbofuran, 3-keto-7-phenolcarbofuran, 3-hydroxy-7-phenolcarbofuran, and 7-phenolcarbofuran. B) The two unidentiﬁed metabolites could be hydroxylated and sulfoxidated derivatives of furathiocarb. Hydroxylation may take place on the carbamate N-methyl group, on an alkyl substituent, or on the aromatic ring itself. SRM: selected reaction monitoring; SCV: sample cone voltage (V); CE: collision energy (eV); RT: retention time.

Furathiocarb and its metabolites concentrations as a function of incubation time were analyzed by LC/MS-MS as depicted in Fig. 3. Mammalian hepatic microsomes (0.15 mg protein/ml) in the presence of NADPH were incubated with 100 µM furathiocarb at 37 ◦C for 20, 40, and 60 min. It is of importance to note that sequential metabolic reactions occur simultaneously during the incubation when a previous metabolite is formed. Thus, the measured amount of a metabolite indicates its concentration only at the time of measurement. Furthermore, distal metabolites may be formed from 2 more proximal metabolites.

In the carbofuran metabolic pathways (Fig. 3 A), it seems that in humanLM further metabolism of carbofuran to 3-hydroxylation and 7-hydroxylation products predominate, whereas in monkeyLM the 7-hydroxylation pathway was less prominent. RatLM showed the highest concentrations of carbofuran (12.7, 15.6, and 18.7 µM, respectively), whereas dogLM produced the lowest (1.7, 0.0, and 0.0 µM, respectively). The lowest concentration of 3-hydroxycarbofuran was measured with ratLM (4.3, 6.1, and 8.2 and µM) and mouseLM (5.2, 9.8, and 12.9 µM) at 20, 40 and 60 min, respectively. Consequently, concentrations of more distal metabolites (3-hydroxy-7-phenol and 3-keto-7-phenol) were the lowest in hepatic microsomes from both rat and mouse. HumanLM, monkeyLM, and dogLM, were the most active in furathiocarb biotransformation to 3-ketocarbofuran and 3-hydroxy-7-phenolcarbofuran for the three incubation times.

Regarding the furathiocarb primary oxidations (metabolite A and B) (Fig. 3B), species differences were quite large, with rat producing the highest amounts and human the smallest amounts, based on the measurements of the first time point 20 min. However, in almost all sets of incubations there was an apparent decline as a function of time, which remains unexplained. It should, however, be noted that no phase II metabolites or any other new metabolite were observed. It is possible that at least a portion of unidentified metabolites are further converted to identified distal carbofuran metabolites and consequently measured in conjunction with these carbofuran pathway metabolites. Both metabolite A and B were consistently highest regarding incubation metabolites formed *via* carbofuran pathways. Both metabolite A and B were conspicuously high and steady in rat and mouse, and human was the lowest, especially regarding metabolite A. It may be of interest that minipig, dog, and rabbit demonstrated quite remarkable declines in the amounts of both peaks. The identification of furathiocarb-oxidated metabolites and their further conversion into other metabolites would need further studies.

The amounts of detected metabolites, except for furathiocarb sulfoxidation/hydroxylation metabolites, were increased by incubation time in the seven mammalian hepatic microsomes in connection to the disappearance of furathiocarb. The aim of metabolic profile characterization was to investigate whether all metabolites detected in humans are also present in the other mammalian hepatic microsomes. However, all metabolites were measured in the seven mammalian species. No species-specific metabolite was found in this study.

To the best of our knowledge, there is no data available on comparative metabolism of furathiocarb in *in vitro* hepatic microsomes nor *in vivo*. However, furathiocarb was detected in human gastric contents and blood of seven fatal poisoning cases and no other metabolites were reported [24]. Only carbofuran was found as a metabolite in an *in vitro* dermal penetration study of furathiocarb with rat abdominal skin using the static diffusion cell. No other metabolites were detected by HPLC [26]. HPLC post-column derivatization-fluorescence detection was employed to detect furathiocarb and three of its metabolites *in vivo* in rats after dermal treatment with furathiocarb [27]. Carbofuran and 3-hydroxy-carbofuran, but not furathiocarb, were detected in plasma and urine after 2, 4, 6, 8, 12, 24, and 48 h of dermal treatments. Carbofuran had the highest concentration in plasma in all time points, while 3-hydroxy-carbofuran concentration was the highest in urine. This finding suggests that 3-hydroxy-carbofuran was more rapidly eliminated in urine due to its high water solubility. However, 3-ketocarbofuran was not detected in plasma or urine [27]. Using the same analytical method, the authors investigated the level of furathiocarb and its metabolites in liver and kidney of the same treated rats. Carbofuran and 3-hydroxy-carbofuran were measured in liver, while only carbofuran was measured in kidney. Furathiocarb was not detected and only traces of 3-ketocarbofuran were detected at 12 h after treatment [28].

In this study furathiocarb and eight metabolites were screened by LC-TOF and quantified by LC/MS-MS. All metabolites that have been identified in rat *in vivo* were also detected in this study. Additionally, some new metabolites, 7-phenolcarbofuran, 3-hydroxy-7phenolcarbofuran, 3-keto-7phenolcarbofuran, and hydroxylated and sulphoxidated furathiocarb, were detected here. In the rat *in vivo* study, only traces of 3-ketocarbofuran were detected at 12 h after treatment. It is possible, due to higher hydrophilicity, that 3-hydroxycarbofuran may be excreted rapidly from rat kidney.

### 3.4 Kinetic analysis of furathiocarb metabolism in hepatic microsomes from various species

The efficiency and affinity of furathiocarb metabolism are essential for characterizing the interspecies differences. In the present study, kinetic parameters of the carbofuran metabolic pathway as well as the sulfoxidation/hydroxylation pathway in hepatic microsomes from various species were investigated using a wide range of furathiocarb (2.5 – 300 µM). The sum of the six metabolites of the carbofuran pathway (carbofuran, 3-hydroxycarbofuran, 3-hydroxy-7-phenolcarbofuran, 3-ketocarbofuran, 3-keto-7-phenolcarbofuran, and 7-phenolcarbofuran) were used for kinetic characterizations, since they are distal metabolites from carbofuran as depicted in Table 1. DogLM had the highest K_m_ value (160.0 µM) indicating the lowest afﬁnity to the enzyme, and the highest capacity corresponding to the highest *V*_*max*_ (37.2 nmol/mg protein/min). MouseLM and ratLM showed the highest and similar afﬁnity (*K*_*m*_ values 25.1 and 24.9 µM), but the capacity was low (*V*_*max*_ values 8.5 and 8.6 nmol/mg protein/min, respectively). HumanLM and monkeyLM had similar capacity (*V*_*m*ax_ values 13.5 and 13.7 nmol/mg protein/min, respectively). The catalytic efﬁciency (*CL*_*int*_) was the highest (443.1 µl/mg protein/min) for monkeyLM, while dogLM had the lowest value (223.8 µl/mg protein/min), about 50% of the highest value. HumanLM, ratLM, mouseLM, and rabbitLM displayed roughly the same catalytic efﬁciency.

**Table 1:** *In vitro* kinetic parameters of formations from carbofuran and furathiocarb oxidation pathways obtained with different mammalian hepatic microsomes and extrapolated hepatic clearance values^1^.

Species	*Sum of carbofuran metabolic pathway (Predominant)*			Sum of furathiocarb oxidation pathways *(minor pathway)*		
	*V*_*max*_	*K*_*m*_	CL_int_	*V*_*max*_	*K*_*m*_	CL_int_
	nmol/(mg protein * min)	µM	µl/(mg protein * min)	nmol/(mg protein * min)	µM	µl/(mg protein * min)
HumanLM	13.5 ± 0.8	40.6 ± 9.6	332.1	2.9 ± 0.6	215.4 ± 102.8	13.6
RatLM	8.5 ± 0.4	25.1 ± 4.5	336.7	7.1 ± 0.9	139.1 ± 45.4	51.4
MouseLM	8.6 ± 0.4	24.9 ± 5.2	345.8	1.3 ± 0.1	120.0 ± 25.9	10.6
DogLM	37.2 ± 1.7	166.0 ± 19.6	223.8	12.8 ± 7.9	1116 ± 629	11.5
RabbitLM	16.1 ± 0.9	47.3 ± 9.7	341.5	8.9 ± 2.4	528.2 ± 244	16.8
MinipigLM	21.7 ± 1.3	53.1 ± 11.3	408.5	11.4 ± 4.4	707.2 ± 315.6	16.2
MonkeyLM	13.7 ± 0.6	31.6 ± 6.1	433.1	10.6 ± 5.1	907.8 ± 425.3	11.6

^1^ Each value represents the mean ± std. error of three determinations.

**Fig. 4:**
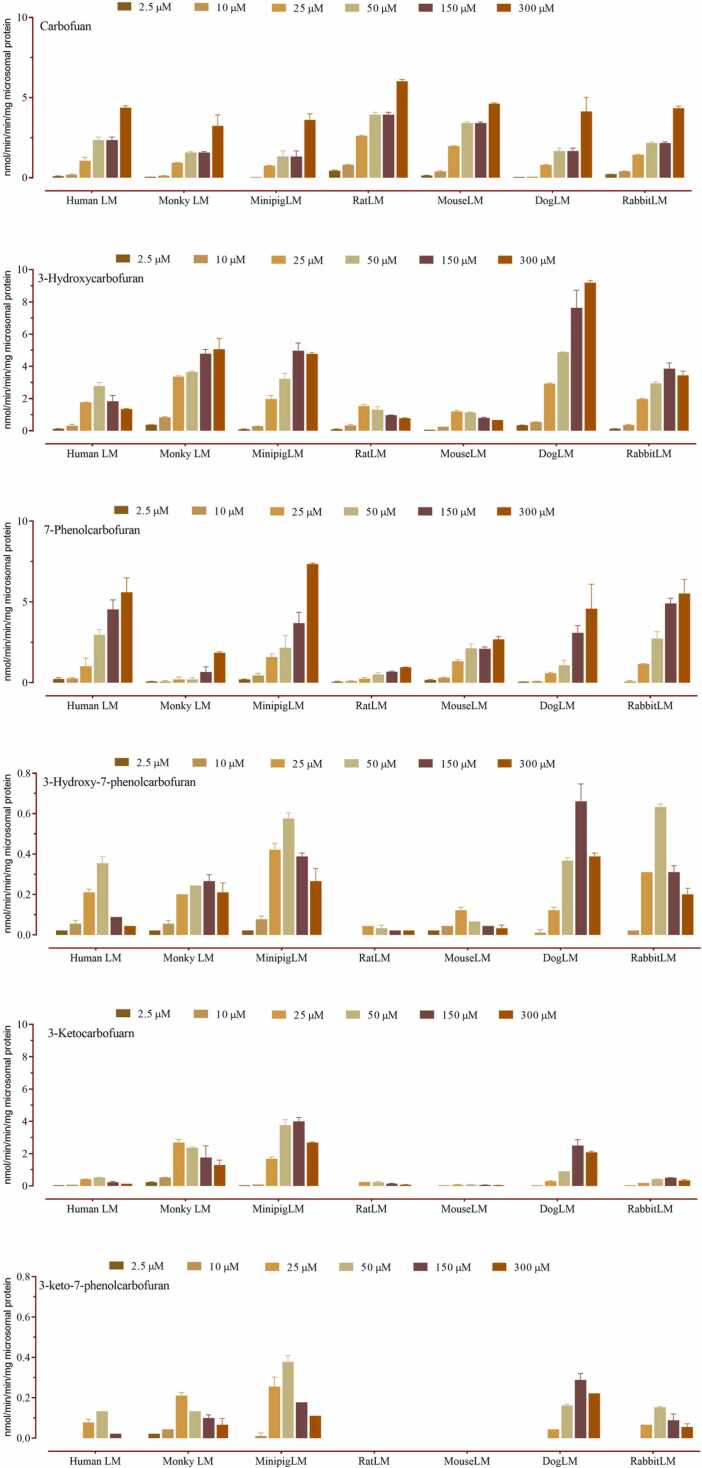
The formation of distal carbofuran metabolic pathway metabolites in the presence of different mammalian hepatic microsomes as a function of furathiocarb concentration. Columns represent the means of three separate measurements and the error bars represent S.D.

Kinetic characterizations of furathiocarb distal metabolites could not be estimated; therefore, metabolite formation rates were used for species comparisons (Fig. 4). The amount of 3-keto-7-phenolcarbofuran formed with mouseLM and ratLM was below the limit of quantiﬁcation. The carbofuran formation rate was roughly the same among the seven hepatic microsomes examined. RatLM had the lowest carbofuran distal metabolites formation rate. HumanLM, ratLM and mouseLM had the lowest 3-hydroxycarbofuran formation rates, while dogLM had the highest formation rate. The rate of 3-hydroxycarbofuran formation was roughly the highest in all mammalian microsomes. Since mouseLM and ratLM had the lowest 3-ketocarbofuran formation rates, its distal metabolite, 3-keto-7-phenolcarbofuran were not detected. MinipigLM displayed roughly the highest formation rates of 3-hydroxy-7-phenolcarbofuran, 3-ketocarbofuran and 3-keto-7-phenolcarbofuran.

Kinetic parameters of the sum of furathiocarb sulfoxidation and hydroxylation metabolic pathways obtained with different mammalian hepatic microsomes and extrapolated hepatic clearance values are shown in Table 1. Three metabolites (carbofuran, 3-hydroxy-carbofuran, 3-ketocarbofuran) observed in carbofuran metabolic pathways are more potent than furathiocarb and its oxidated metabolites [2], [29], [38], [4], [42]. Therefore, hydroxylated and sulfoxidated metabolites were combined to characterize the kinetics for less potent pathway, furathiocarb oxidation pathway. Similar to the carbofuran metabolic pathway kinetics, dogLM had the lowest afﬁnity (*K*_*m*_ = 1116 µM), but the highest capacity (*V*_*max*_ = 12.8 nmol/mg protein/ min) and the reverse was true for mouseLM, which showed the highest afﬁnity, but the lowest capacity (K_m_ = 120.0 µM, *V*_*max*_ 1.3 nmol/mg protein/min, respectively). The *CL*_*int*_ for ratLM (51.4 µl/mg protein/min)) was the highest, while it was the lowest with mouseLM (10.6 µl/mg protein/min), *i.e*. close to a 5-fold difference.

The data from concentration-dependent depletion rate illustrated in Fig. 2 showed a deflection of HLM, MouseLM, and RatLM rates at 150 µM. It is worthy to notice that HumanLM, MouseLM, and RatLM had *K*_*m*_ values ranged between 120 and 215 µM in the furathiocarb oxidation pathway, while other mammalian hepatic microsomes had much higher *K*_*m*_ values ranging from 528 to 1116 µM. On the other hand, *K*_*m*_ values for carbofuran metabolic pathway were much lower than 150 µM, ranging from 24.9 to 53.1 µM, except for DogLM, which had a value of 166.0 µM.

Indeed, there are large variations in physiological parameters between experimental animals and humans. Therefore, in this study, *in vivo* hepatic clearance for both pathways were extrapolated, considering species parameters such as body and liver weight as well as liver blood ﬂow, for better risk translation in humans [11]. *In vitro* as well as *in vivo* extrapolated clearance analyses showed that furathiocarb metabolism to more potent metabolites, the carbofuran metabolic pathway, is dominant (Table 2). The ratios between carbofuran metabolic pathway /furathiocarb oxidation in *in vivo* hepatic clearance were the highest in monkey (9.4-fold), followed by mouse (7-fold), while rat had the lowest differences (2-fold). Species differences were observed in metabolic clearance of another group of pesticides, *i.e*. triazine herbicides, organophosphate insecticides, and carbamates insecticides [23], [3], [4], [41], [43].

**Table 2:** Extrapolated *in vivo* hepatic clearance, and animal to human fold differences.

Species	Sum of carbofuran metabolic pathway (*Predominant / More potent pathway*)		Sum of furathiocarb oxidation pathway *(Minor / Less potent pathway)*		Carbofuran pathway *vs* Oxidation pathway
	*In vivo* hepatic clearance CL_H_ (ml/min)	Animal to human differences (Fold)	*In vivo* hepatic clearance CL_H_ (ml/min)	Animal to human differences (Fold)	Fold
Human	17.9		4.2		4.3
Rat	58.3	3.3	29.4	7.0	2.0
Mouse	69.5	3.9	9.9	2.3	7.0
Dog	31.6	1.8	6.6	1.6	4.8
Rabbit	37.6	2.1	11.2	2.7	3.4
Minipig	35.1	2.0	6.0	1.4	5.8
Monkey	46.6	2.6	4.9	1.2	9.4

In human health risk assessment, a factor of 3.16 is employed as a default uncertainty factor for interspecies differences in toxicokinetics. However, there is no scientific background for this default uncertainty factor [17]. WHO/IPCS recommended utilization of quantitative chemical-specific pharmacokinetic/toxicokinetic data for the development of quantitative toxicokinetic chemical-speciﬁc adjustment factors [12], [45]. Interspecies differences for the carbofuran metabolic pathway, as illustrated by animal to human *CL*_*H*_ comparison (Table 2), showed that mice activate furathiocarb 3.9-fold more than humans, whereas the dog had the closest value (1.8-fold). On the other hand, the interspecies differences for the less potent furathiocarb oxidation pathway showed that rats oxidize furathiocarb 7.0-fold more than humans, whereas the monkey had the closest value (1.2-fold). Because adversities in humans remain mostly undefined just because formal studies in humans cannot be performed, hazard and risk assessment are currently dependent on animal species in toxicological testing. Without scientifically solid tools and procedures it is uncertain to translate *in vivo* and *in vitro* animal toxicological findings into human-relevant information. It is expected that interspecies differences, similar to the ones demonstrated in this paper and elsewhere, would be important inputs into physiologically-based toxicokinetic and toxicodynamic models [20], [33], [37], [39], [5], [7], [9], in which species-selective toxic outcomes are modeled, interpreted and finally, hopefully, predicted and confirmed with targeted studies using new approach methodologies (NAMs) [32].

## 4 Conclusion

This study provided a comprehensive view on qualitative and quantitative similarities and differences of furathiocarb *in vitro* hepatic metabolism in seven mammalian species including human. Furathiocarb was metabolized to 8 detected phase I metabolites. No unique human or experimental animal metabolites were detected. The carbofuran metabolic pathway was predominant than the furathiocarb oxidation pathway in all species studied; differences based on hepatic clearance rates (*CL*_*H*_), were up to 9.4-fold in monkey and 7-fold in rats, while it was 4.3-fold in human. These findings, although restricted to *in vitro* mammalian hepatic metabolism, provide valuable furathiocarb-specific data. Data suggested that interspecies variations, for the potent chemical moiety, are mostly, except for mouse, within the default uncertainty factor for species *in vivo* hepatic clearance. These results are valuable in further defining the uncertainty factors and hazards and risks associated with exposure to furathiocarb.

## Funding

This research was supported by the European Union's Horizon 2020 program EDCMET (grant number 825762).

## CRediT authorship contribution statement

**Khaled Abass**: Conceptualization, Formal analysis, Visualization, Writing – original draft. **Petri Reponen**: Chemical analysis. **Olavi Pelkonen**: Conceptualization, Writing – original draft. All authors: Writing – review & editing.

## Declaration of Competing Interest

The authors declare that they have no known competing financial interests or personal relationships that could have appeared to influence the work reported in this paper.
