# Supplementary material for: Metabolic profiling and in vitro-in vivo extrapolation of furathiocarb in mammalian hepatic microsomes
Source: Toxicol Rep. 2022 Mar 29;9:750–8. doi: 10.1016/j.toxrep.2022.03.030 (PMC9743442; doi:10.1016/j.toxrep.2022.03.030)
Supplement: Supplementary file 1 — Supplementary material [file mmc1.docx]

**Metabolic profiling and *in vitro*-*in vivo* extrapolation of furathiocarb in mammalian hepatic microsomes**

Khaled Abass^1,2,3^*, Petri Reponen^2^, Walaa F. Alsanie^4^, Arja Rautio^1,5^ and Olavi Pelkonen^2^

^1^ Arctic Health, Faculty of Medicine, P.O. Box 7300, FI-90014, University of Oulu, Finland.

^2^ Pharmacology and Toxicology Unit, Research Unit of Biomedicine, P.O. Box 5000, FI-90014 University of Oulu, Oulu, Finland.

^3^ Department of Pesticides, Menoufia University, P.O. Box 32511, Egypt.

^4^ Department of Clinical Laboratory Sciences, The Faculty of Applied Medical Sciences & Centre of Biomedical Sciences Research (CBSR), Taif University, Saudi Arabia.

^5^ Thule Institute, University of the Arctic, FI-90014 Oulu, Finland.

* Corresponding author at: Faculty of Medicine, Arctic Health, University of Oulu, FI-90014 Oulu, Finland.

E-mail addresses: khaled.megahed@oulu.ﬁ, khaled.m.abass@gmail.com (K. Abass).

**MATERIALS AND METHODS**

- 1. **Human liver homogenates and mammalian liver microsomes**

Human liver samples used in this study were obtained from the University Hospital of Oulu as surplus from organ donors. The collection of surplus tissue was approved by the Ethics Committee of the Medical Faculty of the University of Oulu, Finland. Human liver samples were of Caucasian race including 4 female and 6 male subjects between the ages of 21 and 62. Characteristics of the liver samples are presented in Table 1. The livers were transferred to ice immediately after the surgical excision and cut into pieces, snap-frozen in liquid nitrogen and stored at -80 °C. Human liver homogenate was prepared from livers of 10 individuals by homogenizing liver tissue in four volumes of 0.1 M phosphate buffer (pH 7.4), i.e. the homogenate contained 200 mg of liver tissues/ml.

Male DBA/2 mouse, Sprague-Dawley rat, Beagle dog, Cynomolgus monkey, Göttingen minipig, and New Zealand white rabbit liver samples were obtained after approval of the Ethics Board of the Experimental Animal Center of the University of Oulu, Finland. All microsomes were prepared by standard differential ultracentrifugation (Pelkonen et al., 1974). The final microsomal pellet was suspended in 100 mM phosphate buffer, pH 7.4. Protein content was determined by the method of Bradford (Bradford, 1976). It should be noted that this report belongs to a series of articles related to pesticides, including carbamates, biotransformations. Microsomal preparations, cytochrome P450 enzyme activity characterizations and incubations were performed within 12 months during 2007/2008. The initial screening and identification of the metabolites by LC-TOF and quantiﬁcations and fragmentations by LC/MS-MS were carried out over 2008/2009. Data analysis and interpretations as well as publications were performed over prolonged period.

**Table 1. Characterization of the human liver samples.**

| Liver | Age | Sex | Cause of death | Drug history | Liver pathology |
| --- | --- | --- | --- | --- | --- |
| HL20 | 54 | M | ICH | Diazepam^a^ | None |
| HL21 | 44 | M | ICH | Phenytoin^a^, alcohol abuse | Cirrhotic |
| HL22 | 40 | F | ICH | Dexamethasone^a^, nizatidine^a^, phenytoin^a^ | None |
| HL23 | 43 | M | ICH | Diazepam^a^, smoker | None |
| HL24 | 47 | M | ICH | No medication, smoker | None |
| HL28 | 21 | M | Stroke | Dexamethasone^a^, smoker | None |
| HL29 | 39 | F | ICH, SAH | Dexamethasone^a^ | None |
| HL30 | 53 | F | ICH, SAH | No medication | Steatosis |
| HL31 | 44 | F | ICH, SAH | No medication | Steatosis |
| HL32 | 62 | M | ICH, SAH | Metformin, alcohol abuse, smoker | None |

^a^ Drugs were administrated only during the last 24 hours before death; M, male; F, female; ICH, intracerebral hemorrhage; SAH, subarachnoidal hemorrhage; SDH, subdural haematoma.

- 1. ***In vitro* assay and chromatography of furathiocarb metabolites**

The standard incubation mixture contained 100 µM furathiocarb, 0.15 mg pooled liver microsomal protein, and 1 mM NADPH in a final volume of 200 µl of 0.1 M phosphate buffer (pH 7.4). Furathiocarb was prepared once a week in dimethylsulfoxide (DMSO; final amount in the reaction medium 1.0 %). After a 2-min incubation at +37 °C in a shaking incubator block (Eppendorf Thermomixer 5436, Hamburg, Germany), the reaction was started by adding NADPH. The mixture was incubated at +37 °C for 20, 40, and 60 minutes and the reaction was stopped with 600 µl of ice cold acetonitrile containing an internal standard. All incubations were carried out in triplicate. After centrifugation at 10000 × *g* for 15 min, the supernatant was collected and stored at -20 °C until analyzed.

To measure the production of potential metabolites, human liver homogenate incubations were prepared containing the same ﬁnal furathiocarb concentration as the microsomal incubations. In addition to 40 µl of human liver homogenate (contains approximately 0.14 mg microsomal protein), the other components in homogenate incubates were 5 mM uridine 5′-diphosphoglucuronic acid (UDPGA), 1 mM glutathione, 1.2 mM adenosine-3′-phosphate-5′-phosphosulfate (PAPS), and 1 mM NADPH in a ﬁnal volume of 200 µl of 0.1 M phosphate buffer (pH 7.4). The mixture was incubated at +37 ◦C for 20, 40, and 60 min and the reaction was stopped with 600 µl of ice-cold acetonitrile containing an internal standard. The analytical method was similar to the microsomal preparations. Results are expressed as a mean ± standard deviation of three replicates. Enzyme kinetic parameters were measured in microsomal preparations (furathiocarb final concentrations 2.5 – 300 µM). The incubation times were 20 min. Reaction rates were linear at least up to 0.15 mg of microsomal protein/ml and 60 minutes of incubation time.

Chromatographic separation was performed with the Waters Alliance 2690 HPLC system (Waters Corp., Milford, MA) using Waters Atlantis T3 column (2.1 mm x 100 mm, particle size of 3 µm) together with a Phenomenex C18 2.0 mm x 4.0 mm precolumn (Phenomenex, Torrance, CA). The eluent (ultrapure-grade water containing 0.1 % acetic acid (A) and methanol (B)) flow rate was 0.4 mL/min. A linear gradient elution from 5 % B to 75 % B in 8 min was applied. Solvent B was thus maintained at 98 % for 3 min before re-equilibration (6 min).

- 1. **Kinetic parameters**

To measure the enzyme kinetic parameters in microsomal samples, the standard incubation mixture contained furathiocarb (final concentrations 2.5 – 300 µM). Incubation mixtures and methods were the same as mentioned above, except the incubation times were 20 min for microsomal samples. Samples were analyzed by LC-MS-MS. The kinetic parameters *V_max_* and *K_m_* were calculated using Prism 9.0.0 (GraphPad Software, Inc., San Diego, CA) by nonlinear regression. These values were used to calculate the intrinsic clearance value (*V_max_*/*K_m_*). All results are expressed as mean ± standard error for three replicates. In the standard experimental conditions used for furathiocarb metabolites, reaction rates were linear at least up to 0.15 mg of microsomal protein/ml and 60 minutes of incubation time.

- 1. **Mass spectrometry**

The initial screening of the present and accurate mass measurements of compounds were carried out using a Micromass LCT (Micromass, Altrincham, UK) time of flight (TOF) mass spectrometer equipped with a Z-Spray ionization source. A generic positive electrospray ionization method was used for all substrates and metabolites. The capillary voltage was 4000 V, cone voltage 23 V, and desolvation and source temperatures 300 and 150°C, respectively. Nitrogen was used as the desolvation and cone gas with flow rates of 780 and 300 L/h. The mass spectrometer and HPLC system were operated under Micromass MassLynx 3.4 software. For exact mass measurements of metabolites in carbofuran metabolic pathways, the lock mass was *N*-1-naphthylphthalimide ([M+H]^+^ at *m/z* 274.0868), and it was delivered into the ionization source through a T-union using a syringe pump (Harvard Apparatus, Holliston, MA).

The quantification (multiple reaction monitoring, MRM) and fragmentation measurements were performed with a Micromass Quattro II triple quadrupole instrument equipped with a Z-spray ionization source. The capillary voltage was 4000 V, and desolvation and source temperatures 280 and 150°C, respectively. The collision gas was argon with a CID gas cell pressure of 2.0 × 10^3^ mbar. Nitrogen was used as the drying and nebulizing gas with flow rates of 450 and 15 L/h. The selected reaction monitoring (SRM), collision energies, and sample cone voltages for metabolites are presented in Figure 1 in the full text. External standards were measured in the beginning, middle, and end of the experiment to ensure the quality of the analysis. Extracted mass chromatograms of furathiocarb metabolites formed by in vitro incubation with mammalian hepatic microsomes are available from our companion publication submitted in parallel with this report. However, the chromatogram has been added to this manuscript as a supplementary material as well.

Supplementary Figure 1:

Extracted mass chromatograms of furathiocarb metabolites formed by in vitro incubation with mammalian hepatic microsomes. Furathiocarb (ﬁnal concentration 300 µM) was incubated with hepatic microsomes (0.15 mg protein) in the presence of NADPH in 0.1 M phosphate buffer at pH 7.4 for 60 min. 3-hydroxycarbofuran, 3-hydroxy-7-phenolcarbofuran were quantified as the protonated dehydrated molecule [M−H2O+H]+ due to significant in-source fragmentation. Six metabolites (carbofuran, 3-hydroxycarbofuran, 3-ketocarbofuran, 3-keto-7-phenolcarbofuran, 3-hydroxy-7-phenolcarbofuran, and 7-phenolcarbofuran) were identified with the help of authentic standards. Analytical standards were not available for the presumed furathiocarb hydroxylated and sulfoxidated metabolites and they were tentatively identified on the basis of exact masses and fragmentation patterns and quantitated by using the calibration curve of furathiocarb, assuming their responses to be approximately equal. The lower limit of quantitation was 0.5 μM for all compounds. To ensure the quality of the analysis, external standards were measured in the beginning, middle, and end of the experiment. Carbaryl was used as an internal standard (SRM: 202.00 ˃ 145.00; SCV: 25 V; CE: 15 eV; RT: 9.8 min.). SRM; selected reaction monitoring; SCV: sample cone voltage (V); CE: collision energy (eV); RT: retention time. *Hydroxylation may take place on the carbamate N-methyl group, on an alkyl substituent, or on the aromatic ring itself.


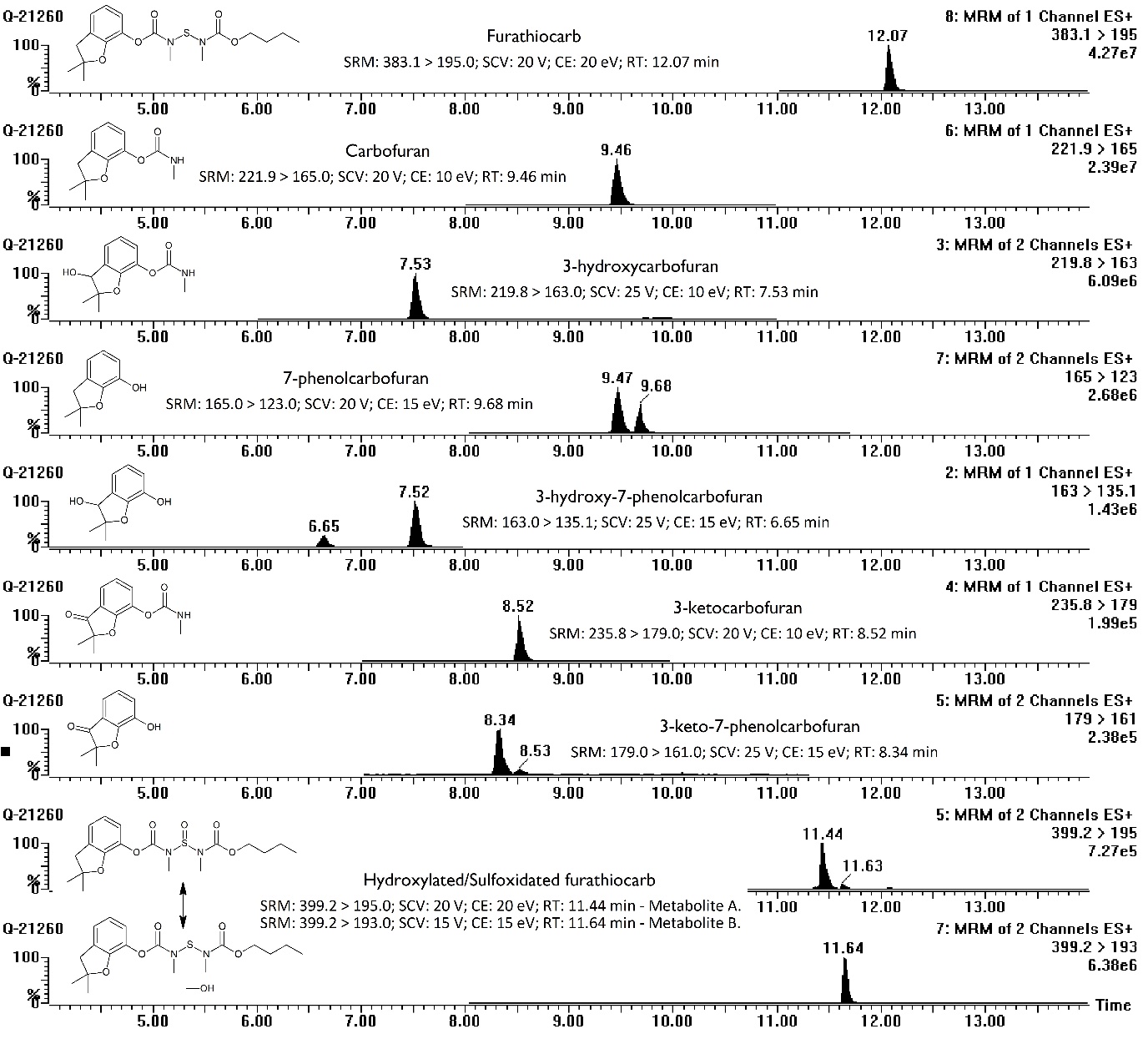


- 1. **The identification and quantification of furathiocarb metabolites**

Analytical standards were available for furathiocarb and carbofuran and its metabolites and the identification was based on the comparison of exact masses and fragmentation patterns of the authentic standards with peaks in the incubates. Quantification was also based on the peak heights of the authentic standards.

Because analytical standards were not available for the presumed furathiocarb hydroxylated and sulfoxidated metabolites, they were tentatively identified on the basis of exact masses and fragmentation patterns and they were quantitated by using the calibration curve of furathiocarb, assuming their responses to be approximately equal. The lower limit of quantitation was 0.5 μM for all compounds. External standards were measured in the beginning, middle, and end of the experiment to ensure the quality of the analysis. Intraday coefficients of variation were less than 20 % throughout the quantitation range of 2.5 –300 μM.

- 1. ***In vitro - in vivo* extrapolations**

*In vivo* for the metabolism of furathiocarb were extrapolated from obtained from incubation with various liver microsomes. *In vivo* hepatic clearances were extrapolated based on *in vitro* data, microsomal protein amount per gram of liver measurements as well as other published values (Barter et al. 2007; Boxenbaum 1980; Pelkonen and Turpeinen 2007) (Table 2).

| Species | ***In vitro – in vivo* extrapolation values** | | | |
| --- | --- | --- | --- | --- |
|  | Body weight (kg) | liver weight (gm) | liver blood flow QH (L/min) | MPPGL^b^ mg/g |
| Human | 70 | 1500 | 1.45 | 18.2 |
| Rat | 0.24 | 10.1 | 0.02 | 23.4 |
| Mouse | 0.03 | 1.54 | 0.003 | 20.6 |
| Dog | 17 | 480.2 | 0.68 | 24.5 |
| Rabbit | 2.9 | 131.5 | 0.112 | 19.5 |
| Minipig | 19.4 | 382^a^ | 0.85^a^ | 22 |
| Monkey | 4.12 | 157 | 0.25 | 12.1 |

**Table 2. Average values of body weights, liver weights, hepatic blood ﬂows and milligram proteins per gram liver (MPPGL).**

^a^ Estimated values based on pig values; ^b^ Experimentally determined values

**REFERENCES**

Barter, Z.E., Bayliss, M.K., Beaune, P.H., Boobis, A.R., Carlile, D.J., Edwards, R.J. et al. Scaling factors for the extrapolation of in vivo metabolic drug clearance from in vitro data: reaching a consensus on values of human micro-somal protein and hepatocellularity per gram of liver. Curr Drug Metab 8:33-45; 2007.

Boxenbaum, H. Interspecies variation in liver weight, hepatic blood flow, and antipyrine intrinsic clearance: Extrapolation of data to benzodiazepines and phenytoin. J Pharmacokinet Pharmacodyn 8:165-76; 1980.

Bradford, A.; rapid and sensitive method for the quantitation of microgram quantities of protein utilizing the principle of protein-dye binding, Anal. Biochem. 72 (1976) 248–254.

Pelkonen, O. and Turpeinen, M. In vitro–in vivo extrapolation of hepatic clearance: Biological tools, scaling factors, model assumptions and correct concentrations. Xenobiotica 37:1066-89; 2007.

Pelkonen, O.; Kaltiala, E.H.; Larmi, T.K.I.; Karki, N.T. Cytochrome P 450-linked monooxygenase system and drug-induced spectral interactions in human liver microsomes, Chem. Biol. Interact. 9 (1974) 205–216.
